# Factors influencing medical students and psychiatry residents in Ghana to consider psychiatry as a career option – a qualitative study

**DOI:** 10.1017/gmh.2020.24

**Published:** 2020-11-03

**Authors:** Vincent I.O Agyapong, Amanda Ritchie, Kacy Doucet, Gerald Agyapong-Opoku, Reham Shalaby, Marianne Hrabok, Thaddeus Ulzen, Akwasi Osei

**Affiliations:** 1 Department of Psychiatry, Faculty of Medicine and Dentistry, University of Alberta, Edmonton, Canada; 2 Alberta Health Services, Edmonton, Canada; 3 St James School of Medicine, Anguilla, Anguilla; 4 Tempo School, Edmonton, Canada; 5 Cumming School of Medicine, University of Calgary, Calgary, Canada; 6 Department of Psychiatry, University of Alabama, Alabama, USA; 7 Ghana Mental Health Authority, Accra, Ghana

**Keywords:** Careers, incentives, medical students, psychiatry, stigma, risk

## Abstract

**Background:**

Currently, Ghana has 14 actively practicing psychiatrists and about 26 psychiatric residents for a population of over 28 million people. Previous research suggests a lack of interest by Ghanaian medical students and medical graduates in considering psychiatry as a career option.

**Objectives:**

To examine the perception of medical students and psychiatry residents in Ghana about the barriers which hinder Ghanaian medical graduates from choosing careers in psychiatry and how these barriers could be overcome.

**Methods:**

This was a cross-sectional qualitative study with data gathered using focus group discussion. Twenty clinical year medical students were selected through block randomization from the four public medical schools in Ghana and invited to participate in one of two focus group discussions. Also, four psychiatric residents were invited to participate in the focus group discussions.

**Results:**

The main barriers identified by participants could be grouped under four main themes, namely: (a) myths and stigma surrounding mental health and patients, (b) negative perceptions of psychiatrists, (c) infrastructure and funding issues, (d) lack of exposure and education. To address the barriers presented, participants discussed potential solutions that could be categorized into five main themes, namely: (a) stigma reduction, (b) educating professionals, (c) addressing deficient infrastructure, (d) risk management, and (e) incentivizing the pursuit of psychiatry among students.

**Conclusion:**

Health policy planners and medical training institutions could consider implementing proposed solutions to identify barriers as part of efforts to improve the psychiatrist to patient ratio in Ghana.

## Introduction

As a medical speciality, psychiatry is growing exponentially to maintain pace with the growing crisis of mental illness worldwide (Wu and Duan, 2015). An estimated 350 million are suffering from depression, which is expected by 2030 to overtake ischemic heart disease worldwide as the number one cause of disease burden (Tucci and Moukaddam, 2017). However, even with this particular speciality in high demand, stigma against mental illness and the practice of psychiatry remains (Bhugra *et al*., 2015). Although residents matching into psychiatry in the United States has increased (Weiner, 2018), the severe shortage continues in low- and middle-income countries (Hailesilassie *et al*., 2017; Warnke *et al*., 2018).

In developing countries, it is estimated that less than 1% of health budgets are spent on community mental health care (Aslam *et al*., 2009). Closely linked with this lack of funding is a lack of interest by medical students to specialize in psychiatry in comparison to the other options available for residency (Aslam *et al*., 2009). Therefore, it is not surprising that many countries are experiencing a shortage of qualified psychiatrists (Lau *et al*., 2015). The lack of psychiatrists in low- to middle-income countries such as Ghana has led to the exploration of several initiatives to address this problem. In Ghana, these initiatives include task shifting to maximize the scope of practice for community mental health workers (Agyapong *et al*., 2015a; Agyapong *et al*., 2015b; 2015c; Agyapong *et al*., 2016a; 2016b; Agyapong, Farren, and McAuliffe, 2016a), and innovative recruitment initiatives focused on mental health workers (Agyapong, 2016) and psychiatrists (Agyapong and McLoughlin, 2012; Agyapong *et al*., 2018).

Given this introduction, the goal of this study is to understand barriers that hinder Ghanaian medical graduates from choosing careers in psychiatry, as examined through the perspectives of medical trainees (i.e. medical students and psychiatry residents). A second goal of the study is to generate solutions to barriers identified by medical trainees, to assist policymakers in achieving improved psychiatrist: patient ratios in Ghana. Specific questions included:

- What are the barriers to choosing a career in psychiatry, from the perspective of medical trainees (i.e. medical students and psychiatry residents)?
- What are the solutions to these identified barriers? What are strategies to encourage Ghanaian medical trainees to pursue a psychiatry specialization?

## Methods

### Study design

This was a qualitative study with data gathered using focus group discussion.

### Study participants and data collection

Twenty clinical year medical students, selected through block randomization from the four public medical schools in Ghana and four psychiatric residents were invited to participate in one of two focus group discussions in Kumasi, Ghana. Both focus groups lasted 3 h, and were moderated by experienced non-physician facilitators. The discussions were recorded and subsequently transcribed verbatim. Focus group questions (online Supplementary Appendix I) explored the barriers to choosing a career in psychiatry from the perspective of the medical trainees. They also explored solutions to identified barriers and strategies to encourage Ghanaian medical trainees to pursue a psychiatry specialization. The medical students and psychiatry residents who participated in the focus group discussions each receive an honorarium to cover the cost of participation, including travel and lodging.

### Data analysis

Participants’ answers to the questions above were transcribed and analyzed thematically using NVivo. The thematic analysis was informed by the methods of Braun and Clarke (2006). The analytic process involved semantic, data-driven coding and a cyclical, reflexive revision process – returning to the data during coding and theming to ensure that the transcripts illustrate the analytic claims and vice versa.

First, structural coding was used to generate initial codes in line with the specific research questions. Pattern coding, which allows identification of explanatory or inferential codes, was further applied to the initial codes to identify patterns or emerging themes and subthemes across the dataset. This second cycle coding used the deductive approach of thematic analysis. Specifically, the generation of the initial themes and subthemes was directed by each of the two overall research goals of the study. The candidate themes and subthemes were further reviewed by checking their ‘fit’ with the collated extracts for each theme and subtheme, as well as with the dataset overall. The final sets of themes and subthemes relevant to the overall research goals were reported and supported with verbatim quotes.

## Results

The participants were asked about factors hindering the accelerated growth in Ghana's psychiatrist population. The main barriers identified by participants could be grouped into four main themes, namely: (a) myths and stigma surrounding mental health and patients, (b) negative perceptions of psychiatrists, (c) infrastructure and funding issues, and (d) lack of exposure and education.

### Myths and stigma surrounding mental health patients

Participants described the stigma that exists in Ghanaian culture as a barrier to their interest in the field. They expressed that stigma manifests as disgust toward people with mental health concerns, referring to them as ‘mad’ and describing them as dirty and aggressive.

*‘Just look at the mad people on our street, wandering about and in dirty, tattered clothes. So if someone says you deal with mad people, then it means you yourself you are dirty.’* – Medical Student

Furthermore, society holds the view that the root cause of mental health issues is spiritual in nature. This adds to the stigma and presents complications in terms of being able to treat the patient.

*‘People subconsciously have it that madness is as a result of some spiritual attack.* [*…*] *it's caused by ‘juju’ or some spiritual force, and that is punishment for some wrong done.’ –* Medical Student

### Negative perceptions about psychiatrists

The cultural views of mental illness in Ghana contribute to the negative perceptions of mental health workers. Psychiatry is not taken seriously – it is considered to be a pseudoscience and lacks the prestige of other medical specialities.

*‘People usually go into a certain field of study because of the prestige it brings cardiologist, neurologist, urologist. Some prestige comes with those names which same cannot be said for psychiatry.’* – Medical Student

### Infrastructure and funding issues

In addition to issues of stigma, the career itself is highly unattractive due to poor funding and infrastructure.

*‘Usually, the structure itself is dilapidated, there is no equipment, even simple beds are lacking, and that is enough to discourage them in addition to the belief and myths they have.’* – Psychiatry Resident

### Lack of exposure and education

Students felt that psychiatry is not often a topic of conversation and therefore, is not considered as a potential career path.

*‘Out of sight, out of mind. If you don't hear about it, you won't think about it so it won't be part of your options’ –* Medical Student.

To address the barriers presented, participants discussed potential solutions in five main themes, namely: (a) stigma reduction, (b) educating professionals, (c) addressing poor infrastructure, (d) risk management, and (e) incentivizing the pursuit of psychiatry among students.

### Stigma reduction

To reduce stigma in the general population, the participants emphasized the importance of increasing the general literacy level in Ghana as well as the need to address the issue of untreated mentally ill people in the streets.

‘*With regards to stigma, I think we would have to tackle it from the root. Pushing for educational reforms.* [*…*] *a pupil knowing that mosquito causes malaria, you see it in your test, so like what causes mental illness?’* – Medical Student

### Educating professionals

Participants recognized the importance of educating professionals and religious leaders so they can work toward collaborating with psychiatrists to treat patients.

*‘In eradicating the cultural and spiritual aspect of stigma, I think one of the people we should be targeting are our spiritual leaders’ –* Medical Student

### Addressing poor infrastructure

Participants recognized that fundraising to address the poor infrastructure for mental health may be a difficult task and so agreed that a better solution might be to integrate psychiatry into existing infrastructure for the time being, and then standalone psychiatric clinics can be a long-term goal.

*‘As the infrastructure is not there, raising money might be a herculean task so we should rather look at integrating it into already existing health care systems. Imagine the nearby hospitals having facilities like that.* [*…*] *rather than pushing for the government to build more hospitals’ –* Medical Student

### Risk management

Risk management must be addressed in order to reduce student's safety concerns.

*‘Hospitals and mental health centres should be better equipped to tackle that risk - emergency medication, sedatives should always be available.’ –* Medical Student

### Incentivizing the pursuit of psychiatry among students

The last solution that was discussed was incentivizing the pursuit of psychiatry among students. Suggested ways of incentivizing the pursuit of psychiatry among students included increasing students’ exposure to the field, improving the student experience and the quality of mental health education, involving diaspora-based psychiatrists as guest lecturers, offering career counselling sessions to medical students and making the job itself more attractive by increasing psychiatrists’ salaries:

*‘Have a separate more enticing salary structure for mental health workers; it will entice students to specialize in psychiatry.’* – Psychiatry Resident

## Discussion

Our study has identified four main barriers hindering medical trainees from pursuing a specialization in psychiatry: (a) myths and stigma regarding mental health, (b) negative perceptions of psychiatrists, (c) infrastructure and funding issues, and (d) lack of exposure and education. To address the barriers presented, participants discussed potential solutions that could be categorized into five main themes, namely: (a) stigma reduction, (b) educating professionals, (c) addressing poor infrastructure, (d) risk management, and (e) incentivizing the pursuit of psychiatry among students.

### Myths and stigma and risk surrounding mental health patients

Patients with mental health conditions are often vulnerable to rejection, discrimination, and lack of access to health care, housing, and employment (Kishore *et al*., 2011). These problems are particularly prominent in low and middle-income countries (Cutler *et al*., 2006; Lau *et al*., 2015; Stuart, 2016). One of the suggestions to combat the stigma that emerged from our study was mental health literacy for Ghanaians, including the incorporation of education in school. Examples of successful educational campaigns exist, such as the World Psychiatric Association's Global Program, which focused on schizophrenia stigma (Stuart, 2016), as well as Mental Health First Aid (MHFA;Kitchener and Jorm, 2002, Stuart, 2016). To address perception regarding risk when working with mental health patients (Stuart, 2016), it is critical to focus training on how to assess risk and mitigate harm appropriately (Slemon, Jenkins, and Bungay, 2017).

### Negative perceptions of psychiatrists

Our study participants identified a negative perception of psychiatrists as a barrier to entry to the profession (Barney *et al*., 2006; Angermeyer *et al*., 2017). It is a common issue for providers to remain pessimistic regarding the likelihood that their patients will recover (Knaak, Mantler, and Szeto, 2017). This leads to an additional barrier, as many practitioners believe that neither their effort nor the effort of their patient will have any effect on the treatment outcome (Knaak, Mantler, and Szeto, 2017).

To address the negative perception of psychiatrists by professional colleagues, our study participants recommend ongoing education, which may be of most utility when delivered via technological means (Clarke, Chambers, and Barry, 2017). Utilization of online resources such as the Hinari Access to Research for Health programme (https://www.who.int/hinari/en/) the WHO-sponsored mhGAP program (https://www.who.int/mental_health/mhgap/en/) and video-based materials (Martin *et al*., 2020) would allow for widespread dispersion of information and would enable information to spread to more rural areas (Clarke, Chambers, and Barry, 2017).

Religious leaders may also play a key role in furthering mental health education to the general public. Studies have shown that religious leaders are often the first direction people go when faced with illness and inability to cope (Pargament and Lomax 2013), and integrating religion and mental health is associated with good outcomes (Pargament and Lomax 2013) and represents a holistic approach to care for some patients (Lake and Turner, 2017).

### Infrastructure and funding issues

Our study participants identified the poor infrastructure of mental healthcare deliver and chronic underfunding as a major barrier to medical students considering psychiatry careers. Mental health care in Ghana is government-funded; receiving 0.5% of the overall health budget, or about 0.007% of gross domestic product (Saxena *et al*., 2006; WHO, 2007). This low budget for mental health affects the training, recruitment, and retention of mental health workers (Agyapong *et al*., 2015a). Spending money on improved mental health care will likely increase the interest of medical students in pursuing psychiatry careers as well as decrease the associated socio-economic costs and the number of years a person spends living with a disability (Lake and Turner, 2017). Consistent with incentives identified by community mental health workers in Ghana (Agyapong *et al*., 2015a), our study participants identified a number of incentive packages that would encourage medical students to venture into the field of psychiatry. This may, thus, be an important focus of governmental policy.

### Lack of exposure and education

Our study participants also identified lack of adequate exposure to psychiatry during medical school as a significant barrier to medical students considering psychiatry careers. Medical students’ perception of psychiatry is heavily influenced throughout their rotations in medical school (Kuhnigk *et al*., 2007; Kuhnigk *et al*., 2009). Key predictors of pursuing a residency in psychiatry included: positive role models, appropriate length and depth of exposure to psychiatry, participation in research, which in turn increased understanding of psychiatry (Bhugra *et al*., 2015; Appleton *et al*., 2017). Students who received exposure to psychiatry during their undergraduate program were more likely to have a positive perception of psychiatry (Lampe *et al*., 2010). Thus, medical schools in Ghana could focus on expanding students’ exposure to psychiatry.

## Limitations of the study

An important limitation to our study is the generalizability of conclusions, given the relatively small sample size.

## Conclusion and policy implications

This is the first qualitative study in Ghana that explores barriers that hinder Ghanaian medical students from choosing careers in psychiatry. Our study extends the literature regarding innovative recruitment initiatives into psychiatry in Ghana (Agyapong and McLoughlin, 2012; Agyapong *et al*., 2018) and has important implications for policymakers.

## Consent for publication

The authors received a consent to publish a declaration from each study participant as part of the general consent for the focus group discussions.

## Supplementary material

For supplementary material accompanying this paper visit http://dx.doi.org/10.1017/gmh.2020.24. click here to view supplementary material

## Data

All transcripts from the focus group workshops are available and can be obtained at a reasonable request from the corresponding author.
