# Supplementary material for: Factors influencing medical students and psychiatry residents in Ghana to consider psychiatry as a career option – a qualitative study
Source: Glob Ment Health (Camb). 2020 Nov 3;7:e31. doi: 10.1017/gmh.2020.24 (PMC7786269; doi:10.1017/gmh.2020.24)
Supplement: Supplementary file 1 [file S2054425120000242sup001.docx]

**Appendix 1**

**Factors influencing medical students and psychiatry residents in Ghana to consider psychiatry as a career option-a qualitative study**

**Focus group discussion questions**

**Barriers to the accelerated growth in Ghana’s psychiatrist population.**

- What are the main barriers that hinder the Ghanaian medical students from considering careers in psychiatry?
- How does stigma influence medical students when considering careers in psychiatry?
- How does consideration about risk associated with working in mental health influence medical students when considering careers in psychiatry?
- How does the infrastructure for mental healthcare delivery and training in Ghana influence medical students when considering careers in psychiatry?
- How does the structure, content and mode of delivery of the curriculum for undergraduate and postgraduate medical education influence medical students when considering careers in psychiatry?
- How does service conditions for mental health workers in Ghana influence medical students when considering careers in psychiatry?

***S*olutions to the barriers to the accelerated growth in Ghana’s psychiatrist population.**

- What are the solutions to the problem of stigma associated with working in mental health so as to encourage more Ghanaian medical students to consider careers in psychiatry?
- What are the solutions to any identified problems of risk associated with working in mental health so as to encourage more Ghanaian medical students to consider careers in psychiatry?
- What are the solutions to any identified infrastructure deficits for mental healthcare delivery and training needed to encourage more Ghanaian medical students to consider careers in psychiatry?
- What are the solutions to problem related to conditions of service for physiatrists needed to encourage more Ghanaian medical students to consider careers in psychiatry?
- What the solutions to problems related to the curriculum content and mode of delivery of the curriculum for undergraduate and post graduate medical education needed to encourage more Ghanaian medical students to consider a career in psychiatry?
- What are the solutions to other identified problems which need to be fixed in other to encourage more Ghanaian medical students to consider a career in psychiatry?
- What other roles should be played by medical schools to encourage more Ghanaian medical students to consider careers in psychiatry?
- What other roles should be played by the Ghana College of Physicians and Surgeons to encourage more Ghanaian medical students to consider careers in psychiatry?
- What other roles should be played by the Government to encourage more Ghanaian medical students to consider careers in psychiatry?

**Impact of diaspora based Ghanaian psychiatrists’ interventions on the growth in Ghana’s psychiatrist population and general mental health services delivery in Ghana and how such interventions could be enhanced**

- How familiar are stakeholders with diaspora based Ghanaian psychiatrists interventions aimed at encouraging more Ghanaian medical students to consider careers in psychiatry?
- Annual inter-medical school public speaking competition now in its seventh year
- Diaspora based Ghanaian psychiatrists’ participation in the teaching of the psychiatric curriculum to Ghanaian medical students and residents.
- To what extent do stakeholders perceive that diaspora based Ghanaian psychiatrists’ interventions have actually encourage more Ghanaian medical students to consider careers in psychiatry?
- To what extent do stakeholders perceive that that diaspora based Ghanaian psychiatrists’ interventions in Ghana have contributed to overall improvement in mental health delivery in Ghana.
- How do stakeholders perceive that diaspora based Ghanaian psychiatrists’ interventions in Ghana could be improved to encourage more Ghanaian medical students to consider careers in psychiatry?
